# Workflow Bottlenecks and Staff Readiness in an NHS Emergency Urology Clinic: A Prospective Service Evaluation to Inform Future AI-Supported Triage

**DOI:** 10.3390/healthcare14111433

**Published:** 2026-05-22

**Authors:** ChingHao Chen, Alice Cotton, Lorin Gresser, Tet Yap

**Affiliations:** 1 Guy’s and St Thomas’ NHS Foundation Trust, London SE1 9RT, UK; alice.cotton@nhs.net (A.C.); tet.yap@nhs.net (T.Y.); 2 DemDx, London EC2Y 9DT, UK; lorin.gresser@demdx.com

**Keywords:** urology, patient flow, workflow analysis, waiting time, digital health, clinical workflow, decision support, NHS

## Abstract

**Background/Objectives:** Efficient patient flow in urgent urology services is critical to timely care delivery, yet workflow bottlenecks in specialty clinics remain underexplored. This study aimed to identify workflow bottlenecks, evaluate patient flow and staff attitudes, and explore clinician readiness for digital decision-support in a high-volume NHS emergency urology walk-in clinic. **Methods:** A two-week observational study was conducted at an emergency urology service in London. Time-stamped pathway data were collected for 80 patient journeys to identify total clinic duration. Differences associated with investigation ordering and senior escalation were analyzed using *t*-tests. Clinicians (*n* = 34) completed a questionnaire assessing perceptions of AI, and nursing staff provided qualitative feedback on operational pressures. **Results:** Mean total clinic journey time was 2 h 42 min, with the post-assessment phase accounting for 64% of total duration. Investigation ordering was the principal source of delay: patients undergoing investigations remained significantly longer in clinic than those who did not (3 h 17 min vs. 2 h 15 min, *p* < 0.05), and doctor-to-discharge time more than doubled (2 h 20 min vs. 1 h 2 min, *p* < 0.005). Senior escalation did not significantly prolong patient flow. Staff surveys demonstrated moderate trust in and comfort with AI as a decision-support tool. Nursing feedback highlighted inappropriate attendances, limited staffing, and workspace constraints as key stressors. **Discussion:** Delays were primarily driven by investigation ordering rather than senior review, identifying investigation timing as a potential target for future pathway optimisation. **Conclusions:** Investigation-related delays were the dominant workflow bottleneck. While no AI system was deployed in this study, these findings provide empirical groundwork to inform the design and prospective evaluation of AI-supported triage in specialty acute care settings.

## 1. Introduction

Efficient healthcare delivery relies on timely patient flow, yet delays within clinical pathways remain a persistent challenge in acute care settings. Artificial intelligence (AI) and machine learning (ML) are increasingly embedded within healthcare delivery, with growing evidence that they can improve workflow efficiency, diagnostic accuracy and clinical decision-making [1,2,3]. AI-supported triage refers to decision-support tools that assist in prioritising patient pathways and early investigation planning, rather than performing diagnostic classification. In acute care settings, this distinction is important, as triage primarily influences patient flow and resource allocation rather than definitive diagnosis. AI-supported triage systems have been implemented across several acute care pathways, including accident and emergency [4,5,6], cardiovascular medicine [7,8,9], ophthalmology [10], obstetrics [11], and patient-led self-triage platforms [12,13]. These systems have demonstrated potential to support prioritisation, reduce waiting times, and optimise clinical flow [2,3,4,5,6,7,8,9,10,11,12,13,14]. However, most existing studies remain early-stage, with limited prospective workflow data and a lack of detailed pathway-level analysis in specialty settings.

Despite progress in other specialties, adoption of AI-assisted triage within urology is scarce. Existing applications of AI in urological care have focused primarily on diagnostic enhancement—such as urinary tract infection detection [15,16], prostate and bladder cancer imaging [17,18,19], diabetic kidney disease assessment [20], and automated urine analysis [21]—rather than operational improvement. No published work has examined how AI could restructure workflow, reduce congestion, or support decision-making in an emergency urology service.

Urology emergency clinics manage diverse presentations ranging from renal colic and acute retention to postoperative complications [22]. This heterogeneity can challenge prioritisation and create delays, particularly in high-volume walk-in services. Triage decisions commonly depend on the judgement and experience of nursing staff and junior doctors, introducing variability that may affect patient flow, waiting time, and resource allocation [14]. Rising demand, workforce pressure, and increasing system congestion have intensified the need for digital tools that improve efficiency without compromising safety [2,23].

National strategy now directly targets this ambition. The NHS Fit for the Future 10-Year Health Plan identifies AI-enabled triage, automated decision-support, and data-driven resource allocation as central to improving productivity, reducing avoidable hospital attendance, and establishing the NHS as the world’s leading AI-enabled health system [24]. To be safely integrated into acute care, AI systems must demonstrate clinical purpose, comparative accuracy, and tangible benefit to patient outcomes or service efficiency. Understanding local workflow, bottlenecks, and user readiness is therefore a necessary first step before design, development, or deployment of AI-supported triage in practice.

Given evidence from other acute specialties that AI-assisted triage can reduce waiting times and streamline pathways, it is reasonable to question whether a similar approach could benefit emergency urology. This study therefore sought to map clinical workflow, identify points of delay, and explore staff attitudes within a London urology walk-in service, to evaluate feasibility, perceived value, and readiness for AI-augmented triage.

## 2. Materials and Methods

### 2.1. Study Design

A prospective observational study was conducted in a high-volume, tertiary referral urology service within a large NHS teaching hospital in London (UK), providing urgent assessment for a broad range of urological conditions. Data were collected over two-week period (23 January–3 February 2023). All adults aged 18 years or older attending during the study period were approached. No patients declined participation, and verbal consent was obtained in all cases. As this project was classified as a service evaluation, participation did not alter clinical care, and refusal did not affect assessment or treatment. No identifiable patient data were collected or stored. No AI platform was used during the service at the time of study, allowing understanding of the clinic’s baseline function to inform feasibility for future AI-supported triage.

The aim of this study was to map the existing clinical workflow, identify bottlenecks, and evaluate the clinical needs and feasibility for the future AI triage system within the service.

### 2.2. The Urology Walk-In Clinic: Existing Pathway

Patients attended via self-referral, referral from the hospital urology team, or their general practitioner. On arrival, patients were registered at reception and completed a paper proforma detailing their presenting complaint and medical history.

Patients were typically seen in order of arrival or appointment time, with exceptions for those presenting with “red flag” symptoms. An on-call nurse then triaged each patient, usually recording vital signs and performing urine dipstick testing. Patients subsequently waited to be reviewed by a junior doctor, who carried out the primary assessment, requested further investigations where indicated (e.g., ultrasound, blood tests, imaging), and made a discharge or admission decision following results. In some cases, patients were escalated to senior clinicians (registrar or consultant) for further review.

Where no additional investigations were ordered, decisions about discharge or admission were made immediately after the doctor review.

### 2.3. Data Collection

Prospective pathway time stamps were collected for each participant, including time of clinic arrival, nurse triage, resident doctor assessment, investigations ordered, and discharge/admission decision (clinic exit) by nurse. Time stamp data were also recorded for patients who required escalation to senior clinicians.

A self-designed five-item questionnaire was distributed to clinic staff (consultants, registrars, resident doctors, and nurses) to capture attitudes towards AI-assisted triage and diagnosis (Appendix A). Items were measured with 10-point Likert scale (1 = Not at all and 10 = Very) with a free-text field for qualitative insights.

Direct verbal feedback was collected from senior nursing staff to identify workflow pressures, operational constraints, and feasibility requirements for implementation of an AI triage system.

Data collection was conducted using a standardised data collection framework by trained members of the research team.

### 2.4. Data Analysis

Time-point data were analysed to determine three phases of clinic throughput: total patient journey time (from arrive to exit); doctor review to clinic exit; and escalation to senior review to clinic exit. Missing data were handled using a complete-case approach and were excluded from the analysis. Data were analysed in Microsoft Excel to calculate means, standard deviations (SD) and ranges. Differences between total journey time for (a) patients where doctors ordered investigations, and (b) patients requiring senior escalation were compared using two-tailed *t*-tests (α = 0.05). F-tests determined whether equal- or unequal-variance *t*-testing was appropriate. Ninety-five percent confidence intervals (95% CI) were generated for mean values.

This study was conducted as an exploratory service evaluation over a defined two-week period and no formal sample size calculation was performed.

To evaluate clinic performance, national and local standards were used as benchmarks. From the time-stamped dataset, the proportion of patients discharged within four hours, the percentage reviewed by a doctor within 30 min (and within one hour), and the frequency with which the clinic closed by 17:00 were calculated and compared against NHS and internal targets.

Questionnaire data were summarised using descriptive statistics. Responses scoring ≤ 5 indicated cautious or negative perceptions; >5 indicated supportive attitudes. Free-text comments and notes from nurse feedback were transcribed, coded, and thematically analysed according to the perceived level of support for AI-assisted triage in the clinic.

### 2.5. Ethical Considerations

Institutional approval was granted as a service improvement evaluation by the Transplant Renal and Urology Digital Board (Project number: 14644 on 18 May 2021). No identifiable patient data were stored or shared externally.

## 3. Results

A total of 80 patient journeys were recorded. Investigation data were available for 51 cases, with 23 patients (45.1%) undergoing at least one investigation. Escalation data were available for 40 cases, of which 28 were escalated to a senior clinician and 12 managed by the resident doctor alone.

### 3.1. Patient Journey Timings

**Figure 1:**
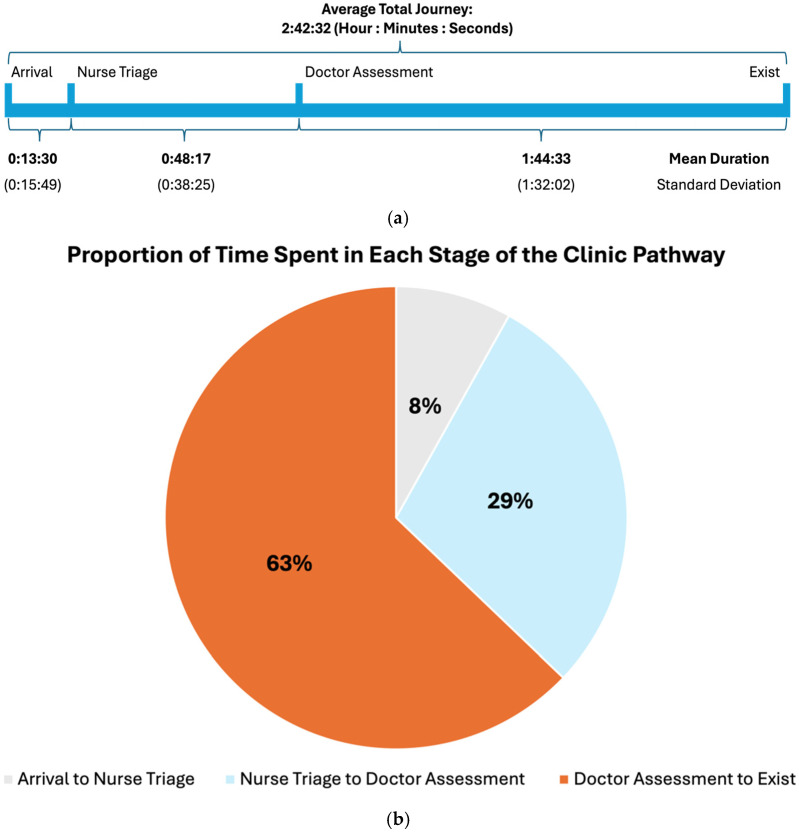

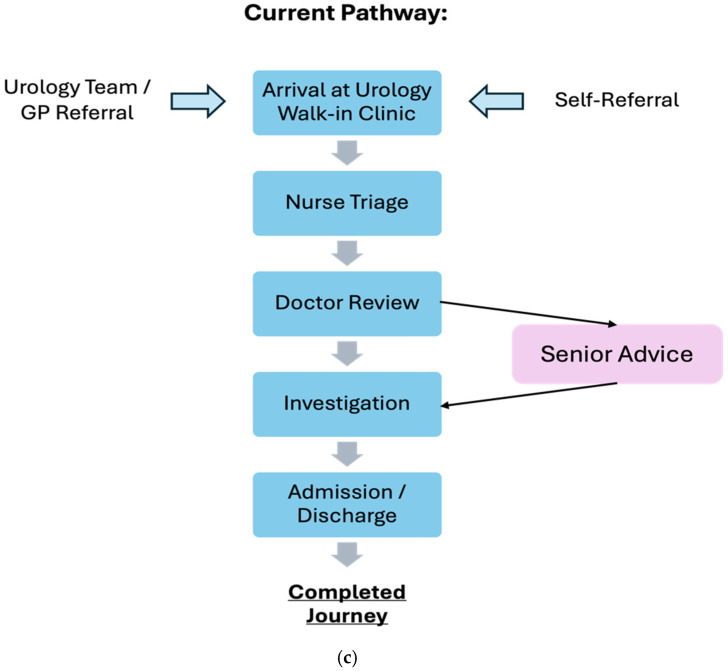
(**a**) Average time of total patients’ journey, (**b**) time distribution, and (**c**) patient journey pathway in the Emergency Urology Walk-in Clinic.

Mean timings intervals for each stage of the clinical pathway are summarised in Figure 1a. The interval from doctor assessment to discharge/admission accounted for approximately 64% of the total journey time, indicating this phase as the largest contributor to clinic waiting duration (Figure 1b). We mapped the workflow of the urology walk-in clinic, outlining the sequential patient journey from arrival through triage, doctor review, investigations, and final disposition, with optional escalation to senior advice in Figure 1c. This workflow highlights that the post-assessment phase, particularly investigation-related processes, represents largest contributor to overall delay.

### 3.2. Impact of Investigations on Patient Journey Time

Total journey time was significantly longer in cases where investigations were requested (Table 1). Patients requiring investigations had a mean total duration of 3 h 17 min (95% CI: 2 h 34 min to 4 h 0 min), compared with 2 h 15 min (95% CI: 1 h 40 min to 2 h 49 min) for those managed without investigations (*p* < 0.05). The between-group men difference was 62 min.

The interval from doctor assessment to clinic exit increased from 1 h 2 min to 2 h 20 min, a 125% increase (*p* < 0.005). Investigations most frequently included ultrasound, CT KUB, blood tests, urine culture, flexible cystoscopy, or X-ray (Appendix B).

### 3.3. Impact of Escalation to Senior Clinicians

Twenty-eight out of forty patients (70.0%) required senior review (Table 1). Reasons for escalation included decisions regarding admission, epididymo-orchitis management, retention, renal stones, suspected bladder cancer, or imaging interpretation (Table 2). Mean total journey time was around 30 min longer when escalation occurred (2 h 36 min vs. 2 h 7 min), but this difference was not statistically significant (*p* > 0.05). Doctor-to-exit duration showed a similar non-significant trend (1 h 34 min vs. 1 h 7 min, *p* > 0.05). The 95% confidence intervals for group means are presented in Table 1.

### 3.4. Clinic Performance Against Targets

Clinic performance fell below expected standards. A total of 65/80 patients (80.8%) were seen and discharged within four hours, against an NHS target of 95%. Review by a doctor within 30 min occurred in 35.5% of cases and within 60 min in 61.8% of cases. These findings suggest inefficiencies concentrated early in the pathway and during post-assessment investigation delays.

### 3.5. Staff Attitudes Towards AI

Thirty-four clinicians completed the AI perception questionnaire (Appendix A). Familiarity with AI was moderately high (mean 7.7/10), while familiarity with AI in urology was lower (4.1/10). Attitudes towards the use of AI in clinical environments were cautiously positive: mean trust in AI for diagnosis and management was 5.7/10, comfort in seeking clinical AI advice was 6.3/10, and support for resident doctors using AI before escalation was 6.1/10 (Appendix C).

Qualitative responses indicated enthusiasm for AI to improve speed and decision-support. However, there were concerns about bias, reliability, accountability, and potential reduced clinical reasoning among junior staff. Respondents generally viewed AI as a supportive tool rather than a substitute for clinical judgement.

### 3.6. Nursing Perspective Barriers to Clinic Flow

Senior nursing staff identified three key operational pressures affecting clinic flow:

1. Inappropriate attendance—A significant number of patients attended with concerns that could have been managed in community settings. This contributed to overall workload, delayed clinically urgent patients, and stretched limited staff capacity.
2. Nursing capacity—Reliance on one nurse for triage and procedures meant that, when the nurse was occupied with catheter care or dressings, triage stalled entirely, delaying flow for all other patients.
3. Infrastructure limitations affecting efficiency—Limited physical workspace and overcrowded staff areas slowed documentation and administrative processing, reducing throughput during busy periods.

While not all infrastructure constraints may be addressable through digital solutions, inappropriate attendances and investigation-related delays were highlighted as areas where AI-supported triage may deliver practical benefit.

## 4. Discussion

This study evaluated workflow patterns, waiting times, and clinical team attitudes in a high-volume urology walk-in clinic to explore where AI-supported triage may add value. The greatest contributor to patient waiting was the period following doctor assessment, accounting for 64% of total pathway duration. The most significant determinant of delay within this phase was the ordering of investigations. Patients requiring investigations remained in the clinic for over an hour longer than those discharged without them, and doctor-to-exit time more than doubled. In contrast, escalation to senior clinicians did not significantly increase pathway duration. Together, these findings suggest that investigation initiation represents a key temporal inflection point within this pathway.

Comparable studies in emergency medicine have demonstrated that AI can reduce triage times, improve prioritisation, and support early risk stratification [25]. While urological emergencies present unique diagnostic pathways, the pattern in our data mirrors results reported in other acute settings: delays occur less in initial triage and more where investigation or imaging is required [2,3,4,5,6,7,8,9,10,11,12,13,14]. These findings suggest that earlier identification and initiation of investigations, particularly at the nursing stage, may represent a potential opportunity to improve pathway efficiency and reduce time spent awaiting tests following doctor assessment. For example, symptom-based prompts (Appendix B) at triage could be explored in future studies to assess whether earlier investigation initiation is feasible and safe.

This is the first study to provide a prospective, time-stamped evaluation of workflow inefficiencies within an emergency urology walk-in clinic, linking objective pathway data with staff perspectives. Rather than evaluating an implemented AI system, this study identifies key operational bottlenecks and proposes a data-informed framework to guide future pathway optimisation.

The integration of workflow timing data and staff perspectives provides complementary insight into service delivery, linking objectively measured delays with perceived operational challenges. This combined approach allows identification of not only where delays occur, but also how they are experienced and managed in clinical practice.

Attitudes towards AI in this study were cautiously optimistic. Familiarity with AI was high, but awareness of AI in urology was lower, reflecting limited clinical deployment in this field. Staff generally supported AI as a decision-support adjunct rather than a replacement, consistent with findings from other healthcare domains where clinicians emphasise the need for oversight, transparency, and diagnostic explainability [3,14,26,27]. The free-text commentary in our survey highlights both the perceived utility (speed, efficiency, reduced unnecessary escalation) and key concerns (bias, accountability, effect on junior learning). Any future implementation must therefore embed safety, interpretability and audit mechanisms, and retain clinician authority as the final decision-maker.

Despite promising signals, structural constraints limit what AI alone can solve. Single-nurse staffing created triage backlogs, and restricted documentation space slowed progress. AI-based triage systems have been proposed in other settings to address non-urgent attendance and investigation sequencing; however, their effectiveness in emergency urology would require prospective validation. Our findings therefore support a hybrid future model in which AI acts not as a throughput solution in isolation, but as a targeted lever within a broader redesign of workforce, space, and demand management [28].

**Figure 2:**
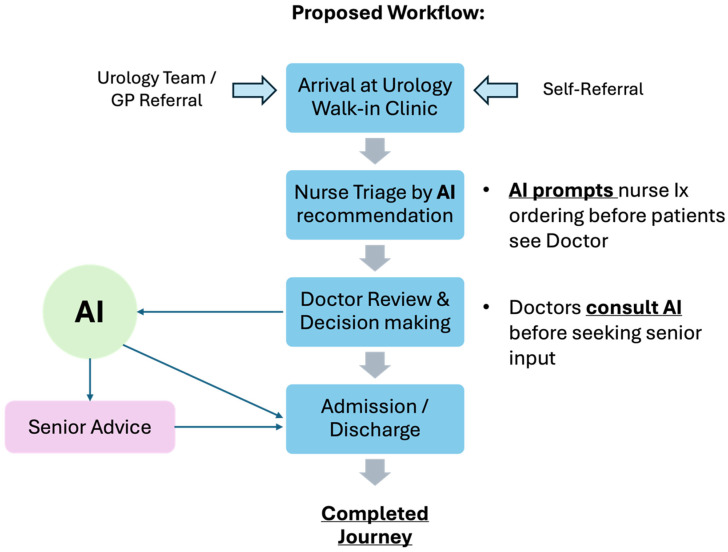
Proposed workflow in the future.

Importantly, a hybrid model may also improve the working experience for clinicians. Evidence from sociotechnical evaluations of AI in clinical settings suggests that, when systems are designed to augment rather than replace human judgement, they may reduce cognitive load, enhance autonomy, preserve problem-solving opportunities, and increase task variety—factors strongly associated with motivation, satisfaction, and retention among healthcare professionals [29]. In this context, AI-augmented triage could support, not replace, clinical decision-making while helping staff redirect time and expertise toward meaningful patient care. Figure 2 outlines a conceptual future workflow derived from the findings of this study. Particularly, the prolonged post-assessment phase observed in our results, largely driven by investigation ordering, highlights a potential opportunity for earlier decision-making within the clinical pathway.

AI-supported triage could potentially assist with early identification of patients requiring imaging, prioritisation of suspected high-risk presentations (e.g., obstructive uropathy, testicular torsion), and decision-support for investigation ordering pathways based on symptom patterns.

The proposed workflow illustrates how digital decision-support could be explored as a potential approach to support earlier investigation decision-making. However, this model is hypothetical and no AI system was implemented in this study. Any such approach would require prospective validation to determine safety, feasibility, and real-world impact before clinical implementation.

### 4.1. Strengths and Limitations

This study provides the first known quantitative evaluation of workflow inefficiencies in an NHS urology walk-in clinic and combines operational data with staff perspectives. However, pathway timing data were limited by missing values and reliance on routine documentation, and escalation data were available for only half of recorded cases. Although no patients were recorded to decline participation, the use of verbal consent without formal documentation may introduce potential selection bias. The sample represents a single centre; so, generalisability may be limited. The two-week observation period reflects a typical operational window for this service; however, seasonal variation and fluctuations in demand may influence workflow patterns. While this study is also limited by a single-centre design and modest sample size, its contribution lies in providing a prospective, time-stamped evaluation of real-world workflow processes, offering detailed pathway-level insight that is not captured in retrospective or outcome-focused studies.

Importantly, no AI platform was implemented or trialled in practice; therefore, results speak to feasibility and anticipated impact based on current workflow patterns rather than direct evidence of clinical performance. We did not collect patient perspectives, which will be essential for future work to ensure usability, acceptability, and ethical deployment of AI-supported triage. Time-based data may be non-normally distributed, and subgroup analyses were limited by modest sample sizes.

Therefore, findings should be interpreted as exploratory and hypothesis-generating rather than evidence of the effectiveness of AI-supported triage.

### 4.2. Implications for Clinical Practice and Future Research

Future development of an AI model for urological triage may prioritise investigation-readiness prediction as a potential area for model development rather than escalation prediction, as this was the primary driver of delay. Prospective evaluation will require co-design with clinicians and patients, integration with EPR systems, and robust validation across multiple centres. Future research should include multi-centre studies, longer observation periods, and repeated sampling across different time points to validate and generalise these findings. A pilot study testing AI-triggered investigation recommendations at triage could quantify impact on waiting time, staff workload, and safety before wider deployment. In parallel, efforts are needed to enhance clinicians’ understanding of AI applications in urology through targeted education and structured training. Understanding both clinical effectiveness and implementation factors will be essential for successful adoption and safe integration into routine urological practice.

## 5. Conclusions

This study demonstrates that delays in the urology walk-in pathway are driven primarily by investigation-related waiting, with escalation to senior clinicians contributing far less to overall time. While no AI system was deployed in this study, the results provide a baseline, data-driven understanding of workflow inefficiencies that may inform the design and prospective evaluation of AI-supported triage in this setting. Future work should involve prototype development, co-design with patients and staff, and prospective testing to determine real-world impact on efficiency, safety, and experience of care.

## Figures and Tables

**Table 1:** Key workflow stress points: impact of investigation ordering and escalation on total journey time.

Total Journey	Mean (95% CI)	SD	*N*	*t*-Test
with Ix Order	3:17:18 (2:34:21–4:00:15)	1:34:22	23	*p* < 0.05
Without Ix Order	2:15:24 (1:40:52–2:49:56)	1:24:41	28	
Total Journey				
With Escalation	2:36:13 (1:59:14–3:13:12)	1:30:40	28	*p* > 0.05
Without Escalation	2:07:40 (1:59:14–3:13:12)	1:21:23	12	
Total Journey from Junior doctor to Exit				
With Ix order	2:20:05 (1:32:33–3:07:37)	1:44:26	23	*p* < 0.005
Without Ix Order	1:02:11 (0:32:10–1:32:12)	1:13:36	28	
Total Journey from Senior doctor to Exit				
With Escalation	1:34:15 (0:55:42–2:12:48)	1:34:32	28	*p* > 0.05
Without Escalation	1:07:30 (0:04:15–2:10:45)	1:34:06	12	

Standard deviation (SD), Confidence interval (CI), Investigations (Ix), hours:minutes:seconds.

**Table 2:** The reason of escalation to a senior doctor and the advice from senior.

Reason of Escalation	Senior Advice
Whether to admit patients	Patient admitted
Resolving epididymitis	Safety Netting Advice and Discharge
Resolving epididymo-orchitis	Safety Netting Advice and Discharge
Chronic urine retention	Patient admitted
Suspected renal stones	Discharged, repeat CT program booked
Suspected bladder cancer	2-week wait, Flexi-cystoscopy, CT KUB
Consultant review of Abdominal X-Ray	Discharged with analgesia
No data available	Safety Netting Advice and Discharge/Discharged with analgesia

**Figure A1:**
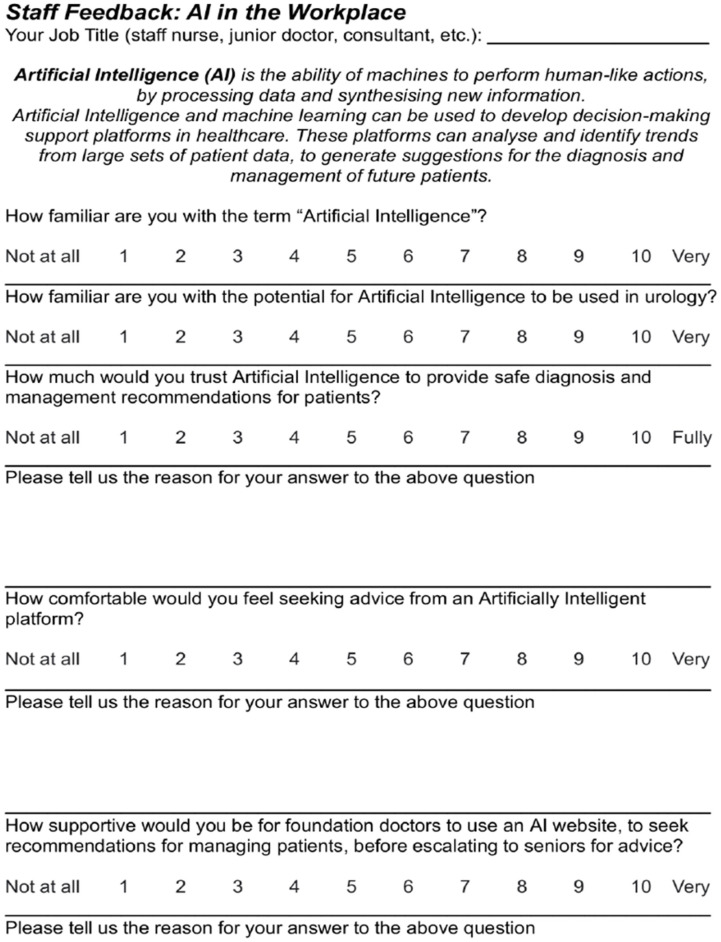
Staff feedback questionnaire.

## Data Availability

The data that support the findings of this study are not publicly available due to privacy and ethical restrictions but are available from the corresponding author upon reasonable request.

## Abbreviations

The following abbreviations are used in this manuscript:

AI	Artificial Intelligence
ML	Machine Learning
NHS	National Health Service
CT	Computed Tomography
KUB	Kidneys, Ureters, and Bladder
PVR	Post-Void Residual
Ix	Investigation

## Appendix B

**Table A1:** A list of investigation. The green highlight in the table represents that the investigations could be ordered by the nurse.

Investigations Ordered by Walk-In Doctors:
Ultrasound: post-void residual bladder, bladder, kidney, testes
CT scan: kidneys, ureters & bladder
Blood tests: prostate-specific antigen, kidney function, full blood counts
Flex Cystoscopy
Midstream urine Culture
Abdominal X-ray
Wound swab (infection)
MRI scan: prostate, kidney, ureter & bladder
Admission

## Appendix C

**Table A2:** Healthcare staff attitudes toward AI in urological practice (*n* = 34).

Question	Mean	Standard Deviation
Q1 How familiar are you with the term “Artificial Intelligence”?	7.66	1.83
Q2 How familiar are you with the potential for AI to be used in urology?	4.14	2.51
Q3 How much would you trust AI to provide safe diagnosis and management recommendations for patients?	5.66	1.99
Q4 How comfortable would you feel seeking clinical guidance or advice from an AI platform?	6.26	2.01
Q5 How supportive would you be for foundation doctors to use an AI website, to seek recommendations for managing patients, before escalating to seniors for advice?	6.14	1.75
